# Course of Vitamin D Levels in Newly Diagnosed Non-Metastatic Breast Cancer Patients over One Year with Quarterly Controls and Substitution

**DOI:** 10.3390/nu16060854

**Published:** 2024-03-15

**Authors:** Cosima Zemlin, Laura Altmayer, Marina Lang, Julia Theresa Schleicher, Caroline Stuhlert, Carolin Wörmann, Laura-Sophie Scherer, Ida Clara Thul, Lisanne Sophie Spenner, Jana Alisa Simon, Alina Wind, Elisabeth Kaiser, Regine Weber, Sybelle Goedicke-Fritz, Gudrun Wagenpfeil, Michael Zemlin, Erich-Franz Solomayer, Jörg Reichrath, Carolin Müller

**Affiliations:** 1 Department of Gynecology, Obstetrics & Reproductive Medicine, Saarland University Medical Center, 66421 Homburg, Germany; cosima.zemlin@uks.eu (C.Z.); laura.alt98@web.de (L.A.); lang.marina@gmx.de (M.L.); juliaschleicher@gmx.de (J.T.S.); caroline.stuhlert@gmail.com (C.S.); carowoer@aol.com (C.W.); laura-sophie.scherer@eurice.eu (L.-S.S.); ida.maier296@googlemail.com (I.C.T.); nelosa@spenner-me.de (L.S.S.); s8jssimo@teams.uni-saarland.de (J.A.S.); alinawind07@gmail.com (A.W.); erich.solomayer@uks.eu (E.-F.S.); 2 Department of Gynecology, Obstetrics & Reproductive Medicine, Saarland University, Campus Homburg, 66421 Homburg, Germany; 3 Department of General Pediatrics and Neonatology, Saarland University Medical Center, 66421 Homburg, Germany; elisabeth.kaiser@uks.eu (E.K.); regine.weber@uks.eu (R.W.); sybelle.goedicke-fritz@uks.eu (S.G.-F.); michael.zemlin@uks.eu (M.Z.); 4 Department of General Pediatrics and Neonatology, Saarland University, Campus Homburg, 66421 Homburg, Germany; 5 Institute for Medical Biometry, Epidemiology and Medical Informatics (IMBEI), Saarland University, Campus Homburg, 66421 Homburg, Germany; gudrun.wagenpfeil@uni-saarland.de; 6 Department of Dermatology, Venereology and Allergology, Saarland University Medical Center, 66421 Homburg, Germany; joerg.reichrath@uks.eu; 7 Department of Anesthesiology, Outcomes Research Institute, Cleveland Clinic, Cleveland, OH 44195, USA

**Keywords:** vitamin D, breast cancer, nutrition, complementary medicine, antitumoral therapy

## Abstract

(1) Background: Vitamin D levels in patients remain inadequately understood, with research yielding inconsistent findings. Breast cancer patients, particularly due to oncological therapies, face an increased risk of osteopenia, which can be exacerbated by a vitamin D deficiency. (2) Methods: The prospective observational “BEGYN-1” study assessed serum 25(OH)D levels at baseline and quarterly thereafter. Clinical, pathological, nutritional, vitamin supplementation, and lifestyle data were recorded. (3) Results: Before treatment, 68.5% of patients were vitamin D deficient (<30 ng/mL), with 4.6% experiencing severe deficiency (<10 ng/mL). The median baseline 25(OH)D levels were 24 ng/mL (range: 4.8 to 64.7 ng/mL). Throughout the study, the median vitamin D levels increased to 48 ng/mL (range: 22.0 to 76.7 ng/mL). Before diagnosis, 16.7% received vitamin D substitution, and 97.8% received vitamin D substitution throughout the year with a median weekly dose of 20,000 IU. It took at least three quarterly assessments for 95% of patients to reach the normal range. A multiple GEE analysis identified associations between 25(OH)D levels and supplementation, season, age, VLDL, magnesium levels, and endocrine therapy. (4) Conclusions: Physicians should monitor 25(OH)D levels before, during, and after oncological therapy to prevent vitamin D deficiency and to adjust substitution individually. While variables such as seasons, age, VLDL, magnesium, diet, and oncological interventions affect 25(OH)D levels, supplementation has the greatest impact.

## 1. Introduction

Breast cancer is the most common malignancy worldwide. In the year 2020, 2.3 million people were diagnosed with breast cancer according to the World Health Organization [1]. It is assumed that the incidence will further increase and by 2040, 3 million people will be diagnosed per year [1]. For this reason, physicians and scientists are constantly working on optimizing prophylaxis, diagnostics, treatment, and therapy strategies. Also, patients themselves want to contribute to the success of treatment. Therefore, the intake of vitamins, micronutrients, or nutritional supplements has become very popular in recent years [2]. However, the effectiveness regarding oncological treatment is considered controversial [2].

One of the most famous vitamins used in this context is vitamin D. It is known that vitamin D deficiency is frequent, with, depending on the source and cut-off values, about 58–77% of German women being affected [3,4]. It can be distinguished between mild, moderate, or severe vitamin D deficiency with serum 25-hydroxyvitamin D (25(OH)D) levels being <30 ng/mL, <20 ng/mL, or <10 ng/mL, respectively [5]. A previous meta-analysis showed the association between 25(OH)D levels and breast cancer prognosis, as well as mortality [6]. Even breast cancer development was linked to 25(OH)D levels in preclinical and animal studies [7,8]. Although, a causal relationship between vitamin D and carcinogenesis in breast cancer patients could not yet be demonstrated in clinical studies [8,9,10].

Vitamin D plays an important role in bone metabolism. Vitamin D deficiency was associated with increased mortality, as well as unfavorable skeletal outcomes, e.g., fractures or bone loss [5]. Additionally, bone metabolism is affected by oncological treatments in breast cancer patients. Of all breast cancer patients, 70–80% suffer from a hormone receptor positive disease and therefore require endocrine therapy [11]. It is known that endocrine therapy with aromatase inhibitors in hormone receptor positive breast cancer patients leads to osteoporosis [12]. Estrogen reduces osteoclast-mediated resorption of the bone and promotes osteoblast-mediated bone formation [13]. With the inhibition of the aromatase enzyme, estrogen levels fall and therefore bone renewal and strengthening are reduced [12]. Furthermore, chemotherapy with anthracyclines and taxanes in breast cancer patients was associated with a worsening of bone mineral density within 6 months and an elevated risk of major osteoporotic fractures at 10 years [14]. In addition, the impact of vitamin D deficiency on therapy-related side effects was shown since patients with paclitaxel chemotherapy suffered polyneuropathy more frequently [15]. As vitamin D deficiency can further deteriorate bone mineral density [5], it is crucial to treat vitamin D deficiency before, during, and after oncological treatment.

The reference range for vitamin D serum level (25(OH)D) is 30–100 ng/mL [16]. Changes in vitamin D levels during oncological treatments were reported previously. Kok et al. observed a decrease in 25(OH)D levels during chemotherapy, with recovery occurring six months after treatment completion [17]. Similar results were observed by Kim et al. [18]. Moreover, they saw an increase in 25(OH)D levels in premenopausal patients with tamoxifen therapy, an estrogen receptor modulator [18].

In the current analysis, we investigated the course of vitamin D levels throughout the first year of newly diagnosed, non-metastatic breast cancer patients. Patients underwent quarterly controls and received substitutions if applicable. We wanted to find answers on how the vitamin D level changed during oncological therapies (e.g., chemotherapy, radiotherapy, endocrine therapy, Her2 targeted therapy), and how vitamin D substitution and lifestyle factors contributed to these changes during the first year after diagnosis of breast cancer.

## 2. Materials and Methods

### 2.1. Data Collection

During September 2019 and January 2021, the prospective observational BEGYN-1 study recruited 110 non-metastatic breast cancer patients at the Saarland University Medical Center. The study was approved by the ethical committee of the Medical Association of Saarland (study # 229/18, date of approval: 11 June 2019). The inclusion criteria were female sex, age ≥ 18 years, invasive non-metastatic breast cancer, sufficient German language skills (to fill out questionnaires and activity diaries), and sufficient technical skills/support to use a smartphone and fitness tracker. Furthermore, every patient needed to sign a written informed consent. Patients were excluded if they received any oncological treatment prior to the baseline assessments, if their life expectancy was less than 12 months, if they had a non-invasive disease (e.g., carcinoma in situ), if they had a current history of other neoplasia, if they were unable to perform a spiroergometry on the treadmill, or if they were pregnant or breast feeding.

**Figure 1:**
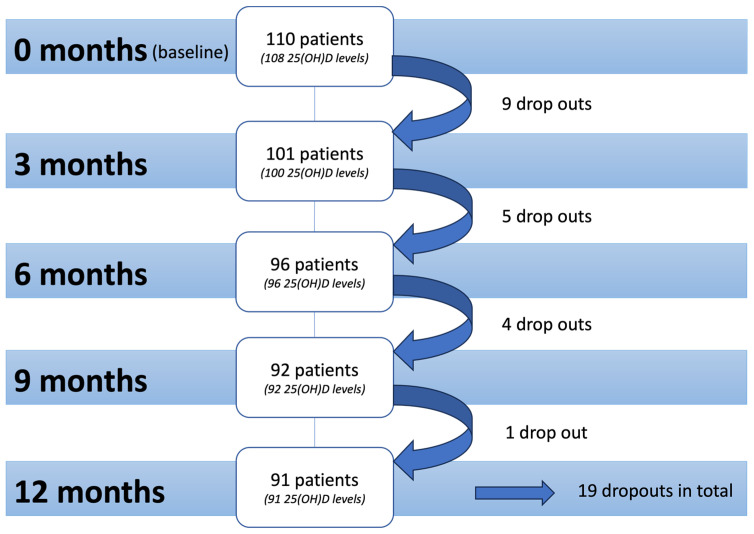
Time of assessments. At baseline assessment (0 months), a total of 110 patients participated in the BEGYN-1 study. Of those patients, 25(OH)D levels were measured of 108 patients. At the final assessment, a total of 91 patients were still in the BEGYN-1 study and received measurement of 25(OH)D levels.

A serum concentration of 25(OH)D was measured using the LIAISON^®^ 25 OH Vitamin D TOTAL Assay (DiaSorin, Saluggia, Italy, REF 310600). The assessments were carried out at the baseline visit (before the start of any pharmacologic, radiation or operative therapy) and then every 3 months throughout the first year after initial diagnosis (Figure 1). The supplementation of vitamins and trace elements was documented at each visit. Patients received supplementation of vitamin D according to their test results and the previously published algorithm for vitamin D dosing by Bleizgys [19]. Most frequently, Cholecalciferol 20,000 IU was prescribed.

Other laboratory values were drawn for all 3 months and analyzed in a routine clinical laboratory. A bioelectrical impedance analysis was performed using the TANITA BC-601 scale ^TM^, Tanita Europe BV, Stuttgart, Germany. Furthermore, information from clinicopathological data was retrieved from the hospital’s digital documentation system (SAP, Walldorf, Germany). Data on sun exposure and nutrition was achieved using a questionnaire at the baseline visit. Data on sun exposure, use of sun protection, and avoidance of going outdoors during “sunny hours” were recently published [10]. Data collection was described in detail previously in the BEGYN study protocol [20].

### 2.2. Statistics

For statistical analyses, SPSS 28.0 (IBM, Armonk, NY, USA) was used. Qualitative parameters are presented as frequencies and quantitative parameters are given as mean with standard deviation or as median with range. The Kolmogorov–Smirnov test was used to test for normal distribution. The association of 25(OH)D levels with lifestyle variables (e.g., age, weight, season of assessment, body composition), laboratory values, and antineoplastic therapy-related factors (e.g., chemotherapy, endocrine therapy, HER2 targeted therapy, radiotherapy, polyneuropathy, tumor marker, polyneuropathy) throughout the year were analyzed using a Generalized Estimating Equations analysis (GEE analysis). Therefore, univariable GEE analysis was performed first. All of the statistically significant variables have been incorporated in a multiple GEE analysis. A multiple GEE analysis was then repeated to prove accuracy. The GEE analysis was employed for parameter estimation in a generalized linear model, accounting for correlations among observations across various timepoints [21].

Some values, such as questionnaires on dietary habits and data on genetic mutation, were only available at the baseline visit. Therefore, linear regression was carried out to analyze an association between baseline vitamin D serum levels and possible influencing factors. Regarding dietary habits, patients who substituted vitamin D were excluded for the analysis. Linear regression was first performed in a univariate analysis. As only one value provided a statistically significant result, no multiple linear regression was performed afterwards.

## 3. Results

At the baseline visit, a total of 110 patients participated in the BEGYN-1 study. Assessments were conducted every 3 months throughout one year after the first diagnosis of breast cancer (0 = baseline, 3, 6, 9 and 12 months, Figure 1). 

A total of 19 patients dropped out of the study. Data on tumor biology and tumor entity were previously published [10]. The median patients’ age was 55 years (min. 26, max. 81). At the baseline assessment, 77 patients (70%) were postmenopausal. Genetic testing was carried out in 42 patients (39%). Of these patients, eight patients (7%) were BRCA1/2 positive, two patients (2%) had a CHECK2 mutation, one patient (1%) had a CHECK3 mutation, and one patient (1%) had an ATM mutation. The occurrence of polyneuropathy was assessed using the Common Toxicity Criteria (CTC criteria), a standardized set used to classify the adverse effects of drugs utilized in cancer therapy [22]. 

**Figure 2:**
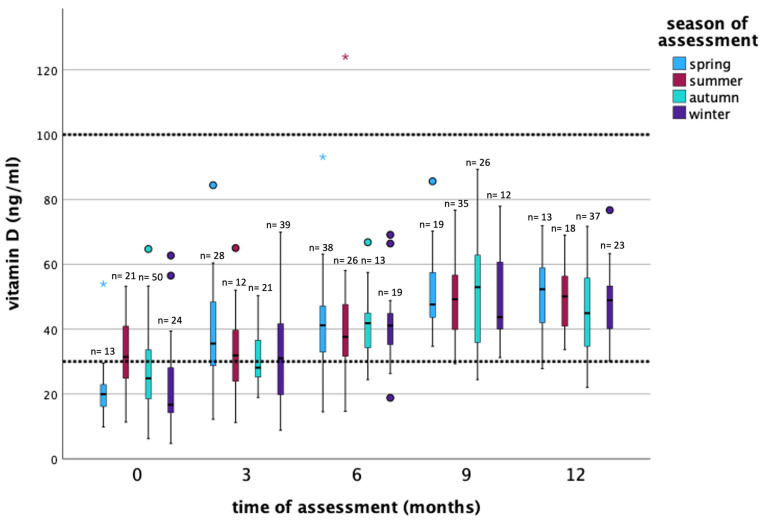
Vitamin D levels in patients dependent on the season of assessment and on different time points (0, 3, 6, 9, 12 months) throughout the study. Vitamin D serum levels referenced in dotted lines (30–100 ng/mL). Dots and stars refer to outliers in the statistical analyses.

Before the initiation of therapy, 68.5% of patients were vitamin D deficient, with a median serum 25(OH)D of 24 ng/mL (min. 4.8, max. 64.7). Throughout the year, median vitamin D level increased to 48 ng/mL (min. 22.0, max. 76.7 ng/mL), Table 1. A total of 18 patients (16.7%) received vitamin D substitution at baseline visit. At the end of the study year, 89 patients (97.8%) needed vitamin D substitution, regardless of the season of assessment (Table 1). The vitamin D levels in patients dependent on the season of assessment and on different time points (0, 3, 6, 9, 12 months) are displayed in Figure 2, as well as in Supplements (Figure S1).

At least three controls of vitamin D serum levels every 3 months were needed until 95% of the patients had 25(OH)D serum levels in the normal range (Table 2). At the fourth control, after 9 months, a total of 89 patients (96.7%) had 25(OH)D levels in the normal range. Of these patients, 86 patients (96.6%) needed to substitute a median of 20,000 IU vitamin D to stay/reach the normal range of 25(OH)D serum levels (Table 2). 25(OH)D levels during different seasons of assessments are displayed in Figure 2 and Supplements (Figure S1).

The data of body composition measured by bioelectrical impedance analysis and laboratory values are displayed in Table 3 and Table 4, respectively. Furthermore, the patients were asked about their dietary habits at baseline visit (Table 5). 

The association of serum 25(OH)D levels with lifestyle variables, as well as antineoplastic therapy related factors, cancer, and side effect factors throughout the year were assessed by multiple GEE analysis. The results are displayed in Table 6. Patients who substituted vitamin D had a higher 25(OH)D serum level (Table 1). When one IU vitamin D was substituted per week, the 25(OH)D levels increased by 0.001 ng/mL (*p* < 0.001). Meaning, a patient who substituted 20,000 IU per week had a medium increase in serum 25(OH)D level of 20 ng/mL compared to a patient who did not substitute vitamin D. Moreover, the season of the assessment showed a significant impact on 25(OH)D levels. In comparison to winter, patients who had their 25(OH)D level determined in spring had on average 3.19 ng/mL (*p* = 0.01) higher 25(OH)D serum level; in summer, 6.66 ng/mL (*p* < 0.001) and in autumn, 4.74 ng/mL (*p* < 0.001), respectively (Figure 2, Table 6). Surprisingly, the age of the patients influenced the 25(OH)D levels positively. If the age increased by one year, 25(OH)D levels increased by an average of 0.15 ng/mL (*p* = 0.03). Furthermore, an increase in VLDL levels and Magnesium levels led to a decrease in 25(OH)D levels throughout the study period. Patients who received aromatase inhibitors as endocrine therapy showed a higher 25(OH)D level compared to patients without endocrine treatment (*p* < 0.001).

In terms of dietary habits, only cream/gouda/butter provided a statistically significant result (Table 7). Patients who ate one day more often cream/gouda/butter per month had an average of 0.38 ng/mL lower 25(OH)D levels at baseline. 

## 4. Discussion

Vitamin D deficiency is a very frequent problem. In a pooled estimate of 55,844 participants in Europe, 13.0% of European individuals had serum 25(OH)D concentrations of <12.03 ng/mL (corresponding to 30 nmol/L) [24]. According to data from the German Nutrition Society, 76.9% of German women between 18 and 79 years of age suffered a vitamin D deficiency (<30 ng/mL), and even 17% suffered from a severe vitamin D deficiency (<10 ng/mL) in 2011 [4]. In the BEGYN-1 study, while the number of patients experiencing vitamin D deficiency was not as high, a notable percentage of all participants still exhibited this condition (68.5% patients (<30 ng/mL) and 4.6% of patients suffered from severe deficiency (<10 ng/mL)). The difference could be primarily due to a general change in health awareness over the last 10–15 years and a shift from indoor to outdoor activities, especially during the COVID-19 pandemic while the study took place [25,26]. Nevertheless, the rate of vitamin D deficiency at the baseline assessment of the BEGYN-1 study was still very high, with a median serum 25(OH)D level of 24 ng/mL and therefore below the recommended minimal level of 30 ng/mL [16]. As oncological treatments like chemotherapy and endocrine therapy aggravate issues in bone metabolism, ensuring adequate vitamin D levels for breast cancer patients is crucial for the prevention of osteoporosis and the benefit of bone health [12,14]. For this reason, routine control, as well as the adequate substitution of vitamin D and calcium during oncological therapies, especially during endocrine therapy with aromatase inhibitor or ovarian function suppression, is recommended by national and international oncological guidelines [27,28]. 

**Figure 3:**
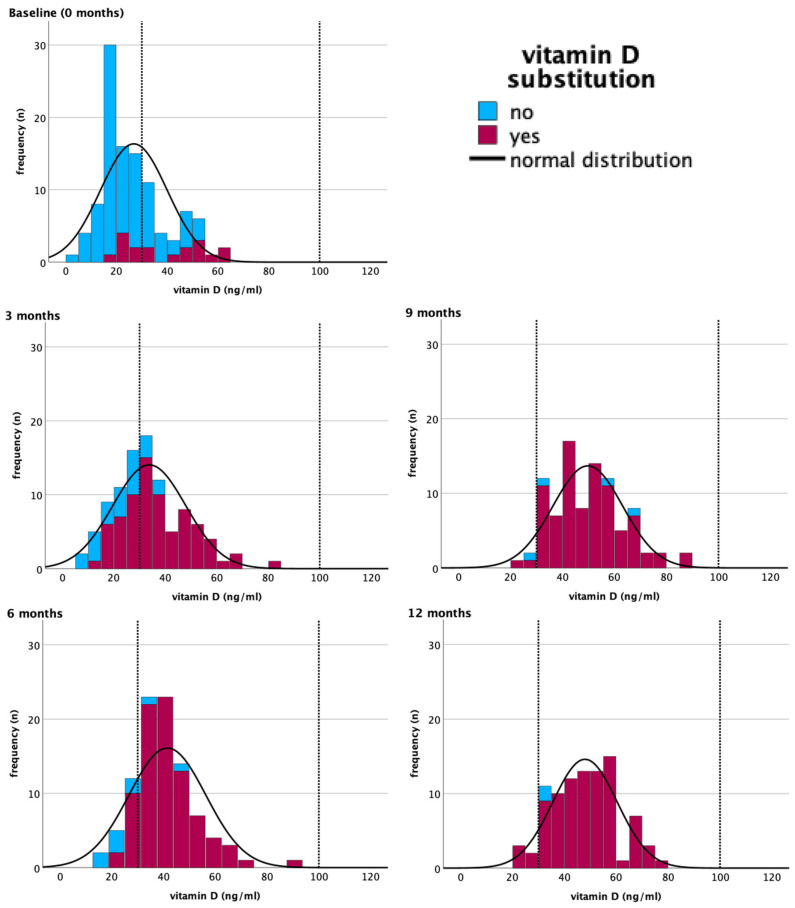
Vitamin D serum levels throughout the year (0, 3, 6, 9, 12 months) with and without substitution. Reference range in dotted line (30–100 ng/mL).

Furthermore, serial monitoring of 25(OH)D levels is recommended by the Endocrine Society Guidelines in patients who substitute vitamin D [29]. According to the European Expert Consensus, at least one control after 6 to 12 weeks of substitution should take place to ensure adequate vitamin D levels and prevent hypercalcemia in patients with substitution [30]. During the BEGYN-1 study, it became evident that additional measurements were required to ensure sufficient serum 25(OH)D levels in certain individuals (Figure 3). We saw that at least three controls every 3 months were needed until more than 95% of the patients reached/stayed in the normal range for 25(OH)D serum levels. Regarding the dosage, a median of 20,000 IU vitamin D was necessary to raise 25(OH)D levels into the normal range in the BEGYN-1 study. We demonstrated that the 25(OH)D levels increased regardless of their baseline level by a median of 20 ng/mL, if patients substitute 20,000 IU vitamin D per week throughout the year. This roughly lies within the recommendations of the Endocrine Society Clinical Practice Guidelines; they recommend a substitution of 50,000 IU of vitamin D_2_ or D_3_ for patients with a vitamin D deficiency (<20 ng/mL) once a week for at least 8 weeks, followed by a maintained substitution of 10,500–14,000 IU per week, as soon as the patients achieve the normal range for vitamin D levels [29]. However, in patients with risk factors of vitamin D deficiency, such as malabsorption or medications affecting the vitamin D metabolism, required doses can be two to three times higher [29]. In the BEGYN-1 study, there were also patients that required much less (2000 IU/week) or more (60,000 IU/week) vitamin D substitution to achieve the recommended normal range (30–100 ng/mL). As every patient is different, lifestyle factors such as sun exposure [31], nutrition [16,23], BMI [32], age [33,34], and vitamin D receptor genetics [35] play a role in 25(OH)D levels. This further illustrates that a “one size fits all” approach is ineffective, and that regular monitoring is necessary [36].

The major source of “natural” vitamin D is the exposure of the skin to sunlight; therefore, 25(OH)D levels were lowest during winter months [29,37,38]. These results were confirmed in the BEGYN-1 study. Even after the consideration of other factors like substitution and lifestyle, a noteworthy seasonal impact was observed, leading to decreased vitamin D levels during the winter months (median serum 25(OH)D levels were 6.66 ng/mL lower in winter compared to summer, *p* < 0.001). Therefore, the season of assessment should also be considered during regular monitoring. Moreover, we previously demonstrated higher 25(OH)D levels in non-metastatic breast cancer patients who stated that they stayed longer in the sun [10]. 

Previous studies showed higher rates of vitamin D deficiency in older patients [33]. However, not only does the age-dependent reduced synthesis of vitamin D in the skin play a role, but also the increased rate of hospitalizations, immobilization, increased rate of renal insufficiency, and the intake of various medications might affect vitamin D metabolism [33,34,39,40]. While there have been suggestions that the aging process also might diminish the intestine’s capacity to absorb dietary vitamin D, research has shown that aging does not affect the absorption of both physiological and pharmacological doses of vitamin D [41,42]. In the BEGYN-1 study, patients who were older had slightly higher vitamin D levels throughout the year. One factor contributing to this might be that the median age of the patients was 55 years, indicating that they are not inherently considered “old”. Another factor could be the increased awareness of vitamin D deficiency, leading older patients to potentially counteract low 25(OH)D levels [43]. Especially during retirement, older individuals tend to spend more time outdoors throughout the week compared to younger, employed individuals, resulting in higher vitamin D levels [44].

Vitamin D is a fat-soluble vitamin [32,45]. For this reason, obese adults (BMI > 30 kg/m^2^) might be at a higher risk for vitamin D deficiency, as the hormone is stored in fatty tissues [32,45]. In the BEGYN-1 study, we could not prove a significant association between 25(OH)D levels and weight, BMI, as well as body fat. We have no explanation, therefore. However, we saw that elevated VLDL levels were associated with lower 25(OH)D levels. The relationship between 25(OH)D levels and lipid profile was prescribed previously [46]. We further saw an association between 25(OH)D levels and magnesium. Magnesium is involved in vitamin D metabolism and plays an important role in both activation and deactivation [47]. Results from randomized controlled trials even showed that magnesium substitution next to vitamin D substitution may be more effective than vitamin D alone [48,49]. One step in the magnesium–vitamin D metabolism is the transformation from 25(OH)D to 1,25(OH)_2_D, as well as 24,25(OH)_2_D [47]. A possible explanation for our reversed association between magnesium and vitamin D could be that the increased supply of magnesium led to an increased metabolism of 25(OH)D and therefore to reduced serum levels.

Regarding oncological therapies, previous studies showed a decrease in 25(OH)D levels during chemotherapy [17,18]. However, the 25(OH)D levels recovered after the end of chemotherapy treatment [17,18]. A comparable observation was made in the BEGYN-1 study. Descriptively speaking, patients receiving chemotherapy had a lower median 25(OH)D level after 3 months compared to patients without chemotherapy (30.5 ng/mL vs. 35.9 ng/mL). Nevertheless, when considering a one-year-long timeframe, and accounting for other variables such as substitution and seasonal variations, chemotherapy did not demonstrate a significant impact on the 25(OH)D level after one year. Yet, a noteworthy association emerged among patients undergoing endocrine therapy, specifically those on aromatase inhibitors. In comparison to those not undergoing endocrine therapy, these individuals exhibited an average 25(OH)D level that was 8.58 ng/mL higher (*p* < 0.001). This contrasts with the current literature. As explained in the introduction, the use of aromatase inhibitors was associated with osteoporosis and higher rates of bone fractures [50]. Furthermore, vitamin D demonstrated a tissue-selective aromatase inhibitor action and could also play a role in modulating aromatase inhibitor-related osteoporosis [51]. Vitamin D and calcium substitution, bone density measurement, and adequate patient education during therapy with aromatase inhibitors are therefore already part of the clinical routine due to national and international guidelines [27,28]. For this reason, we assume that the improved 25(OH)D levels observed in patients receiving aromatase inhibitor treatment in the BEGYN-1 study can be attributed to this circumstance.

Regarding nutrition, we only saw an association between 25(OH)D levels and cream/gouda/butter. However, this effect seems clinically negligible considering a decrease of 0.38 ng/mL in a total range of 30–100 ng/mL of “normal” vitamin D levels if the patients consumed cream/gouda/butter one more day per month. In the literature, the impact of consuming foods rich in vitamin D on the 25(OH)D serum level has also been described as minimal compared to substitution and sun exposure, accounting for approximately 10–20% of the vitamin D content present in the body [16]. The nutritional influence could be increased by the fortification of food [52,53]. However, in Germany, fortification is currently not mandatory, unlike in the United States [54]. Therefore, the prevalence of severe vitamin D deficiency (<10 ng/mL) is only 2.6% in the United States [55].

In contrast to vitamin D deficiency, an oversupply of vitamin D is extremely rare. In the BEGYN-1 study, only one patient had an overdosage of vitamin D at the assessment at 6 months (124 ng/mL). Yet, attaining such an elevated serum level of 25(OH)D is not possible solely through exposure to sunlight [16,56]. The excessive intake of an external vitamin D supplementation is the primary cause of vitamin D hypervitaminosis, with only a minor portion potentially attributed to the internal dysregulation of vitamin D metabolism [56]. In the BEGYN-1 study, the patient self-administered 140,000 IU of vitamin D weekly—against the physicians’ advice. This underscores the significance of routine monitoring of 25(OH)D levels to avert potential complications associated with vitamin D overdose, such as hypercalcemia and its symptoms (confusion, apathy, muscle weakness, abdominal pain, dehydration, polyuria) [56,57].

The limitations of this study are that supplementation and nutrition habits are reported by the patients themselves. Therefore, we had to rely on patients’ compliance. Furthermore, the patients took different vitamin D supplements. This could also have had an impact on 25(OH)D levels, due to the different bioavailability of vitamin D [58].

## 5. Conclusions

Despite oncological therapy, it is possible to increase and maintain normal 25(OH)D levels. This requires regular checks and if necessary, adequate substitution to maintain the recommended 25(OH)D levels. The season of assessment, lifestyle factors (e.g., nutrition), age, and oncological therapy impact the vitamin D levels. However, the biggest effect on 25(OH)D levels is the substitution of vitamin D. At least three controls every 3 months were necessary, such that more than 95% of patients had a 25(OH)D level within normal range. Additionally, substitution should be monitored and adjusted accordingly before, during, and after oncological treatment to ensure normal 25(OH)D serum levels and to avoid overdosage or persisting deficiency.

## Figures and Tables

**Table 1:** Serum 25(OH)D levels in ng/mL throughout the year (0, 3, 6, 9, 12 months), depending on seasons with or without substitution.

Time of Assessment	Substitution	Frequency *n* (%)	Mean ng/mL (±SD *)	Median ng/mL (Min–Max **)	Season of Assessment *** n (%)
0 months (baseline)	no	90 (83.3%)	24.3 (±11.1)	21.5 (4.8–53.2)	SP = 11 (12.2%) SU = 19 (21.1%) AU = 41 (45.6%) WI = 19 (21.1%)
	yes	18 (16.7%)	39.4 (±15.7)	38.1 (15.7–64.7)	SP = 2 (11.1%) SU = 2 (11.1%) AU = 9 (50%) WI = 5 (27.8%)
	all patients	108 (100%)	26.8 (±13.2)	24.2 (4.8–64.7)	SP = 13 (12.0%) SU = 21 (19.4%) AU = 50 (46.3%) WI = 24 (22.2%)
3 months	no	24 (24%)	22.7 (±9.1)	22.7 (8.8–39.4)	SP = 4 (16.7%) SU = 3 (12.5%) AU = 5 (20.8%) WI = 12 (50%)
	yes	76 (76%)	37.3 (±13.8)	34.4 (11.9–84.4)	SP = 24 (31.6%) SU = 9 (11.8%) AU = 16 (21.1%) WI = 27 (35.5%)
	all patients	100 (100%)	33.8 (±14.2)	32.7 (8.8–84.4)	SP = 28 (28%) SU = 12 (12%) AU = 21 (21%) WI = 39 (39%)
6 months	no	9 (9.4%)	25.9 (±11.0)	23.2 (14.5–47.6)	SP = 4 (44.5%) SU = 3 (33.3%) AU = 0 (0%) WI = 2 (22.2%)
	yes	87 (90.6%)	43.1 (±14.3)	41.3 (20.9–124.0)	SP = 34 (39.1%) SU = 23 (26.4%) AU =13 (14.9%) WI = 17 (19.6%)
	all patients	96 (100%)	41.5 (±14.9)	40.8 (14.5–124.0)	SP = 38 (39.6%) SU = 26 (27.1%) AU = 13 (13.5%) WI = 19 (19.8%)
9 months	no	4 (4.3%)	46.4 (±18.3)	44.9 (29.3–66.5)	SP = 1 (25.0%) SU = 1 (25.0%) AU = 2 (50.0%) WI = 0 (0%)
	yes	88 (95.7%)	49.8 (±13.3)	49.0 (24.4–89.3)	SP = 18 (20.5%) SU = 34 (38.6%) AU = 24 (27.3%) WI = 12 (13.6%)
	all patients	92 (100%)	49.7 (±13.4)	49.0 (24.4–89.3)	SP = 19 (20.7%) SU = 35 (38.0%) AU = 26 (28.3%) WI = 12 (13.0%)
12 months	No	2 (2.2%)	32.2 (±2.2)	32.2 (30.6–33.7)	SP = 0 (0%) SU = 1 (50.0%) AU = 1 (50.0%) WI = 0 (0%)
	yes	89 (97.8%)	48.3 (±12.3)	48.7 (22.0–76.7)	SP = 13 (14.6%) SU = 17 (19.1%) AU = 36 (40.5%) WI = 23 (25.8%)
	all patients	91 (100%)	47.9 (±12.4)	48.1 (22.0–76.7)	SP = 13 (14.2%) SU = 18 (19.8%) AU = 37 (40.7%) WI = 23 (25.3%)

* SD = standard deviation, ** min–max: minimum–maximum of median, *** season of assessment: SP = spring, SU = summer, AU = autumn, WI = winter.

**Table 2:** Serum 25(OH)D levels throughout the year (0, 3, 6, 9, 12 months) differentiated between vitamin D deficiency, normal range, and overdosage. The numbers are given in number of patients (n) and percentage (%). Furthermore, vitamin D substitution in IU per week is displayed with median (minimum–maximum) in patients with normal range 25(OH)D levels.

Time of Assessment	0 Months (Baseline)	3 Months	6 Months	9 Months	12 Months
**Vitamin D serum levels**					
Total Vitamin D deficiency (<30 ng/mL)	74 (68.5%)	43 (43%)	14 (14.6%)	3 (3.3%)	5 (5.5%)
Vitamin D normal range (30–100 ng/mL)	34 (31.5%)	57 (57%)	81 (84.4%)	89 (96.7%)	86 (94.5%)
Vitamin D overdosage (>100 ng/mL)	0	0	1 (1%)	0	0
Total	108 (100%)	100 (100%)	96 (100%)	92 (100%)	91 (100%)
**Vitamin D deficiency**					
Severe vitamin D deficiency (<10 ng/mL)	5 (4.6%)	2 (2%)	0	0	0
Moderate vitamin D deficiency (10–19.9 ng/mL)	38 (35.2%)	14 (14%)	4 (4.2%)	0	0
Mild vitamin D deficiency (20–29.9 ng/mL)	31 (28.7%)	27 (27%)	10 (10.4%)	3 (3.3%)	5 (5.5%)
**Vitamin D normal range**					
Vitamin D normal range (30–100 ng/mL)	34 (100%)	57 (100%)	81 (100%)	89 (100%)	86 (94.5%)
How many patients substituted to have normal range	11 (32.4%)	52 (91.2%)	78 (96.3%)	86 (96.6%)	84 (97.7%)
Vitamin D substitution in patients with normal range (IU/week) *	20,000 IU (2000–40,000 IU)	20,000 IU (2000–140,000 IU)	20,000 IU (2000–60,000 IU)	20,000 IU (2000–140,000 IU)	20,000 IU (5600–140,000 IU)

* As values were not normally distributed, numbers are given as median with minimum and maximum.

**Table 3:** Body composition. Body composition (by bioelectrical impedance analysis) over the course of the study year (0, 3, 6, 9, 12 months). As most values were not normally distributed by Kolmogorov–Smirnov test, all values are given as median with minimum and maximum value.

Time of Assessment	0 Months (Baseline)	3 Months	6 Months	9 Months	12 Months
Weight (kg)	69.6 (45.6–107.4)	68.5 (43.9–103.4)	68.6 (44.5–106.1)	68.7 (43.6–107.0)	69.2 (45.4–108.4)
BMI (kg/m^2^)	25.7 (19.2–39.0)	25.7 (18.8–38.0)	25.7 (18.9–37.5)	25.8 (17.9–37.5)	25.7 (18.6–38.0)
Body fat (%)	34.9 (16.9–48.5)	33.1 (17.0–48.2)	32.0 (17.1–46.1)	33.8 (19.1–47.1)	34.2 (17.3–46.4)
Muscle mass (kg)	43.6 (34.4–55.8)	43.5 (33.8–63.2)	43.9 (32.4–59.4)	43.7 (30.9–56.0)	43.2 (31.6–59.4)
Bone mass (kg)	2.3 (1.9–3.0)	2.3 (1.8–4.6)	2.4 (1.8–3.2)	2.3 (1.7–3.0)	2.3 (1.7–3.2)
Visceral fat (kg)	7.0 (2.0–15.0)	7.0 (2.0–17.0)	7.0 (2.0–13.0)	7.0 (2.0–14.0)	7.0 (3.0–14.0)

**Table 4:** Laboratory values. Blood lipids, thyroid hormones, HbA1c, minerals, trace elements over the course of the study year (0, 3, 6, 9, 12 months). As values were not normally distributed by Kolmogorov–Smirnov test, all values are given as median with minimum and maximum value.

Time of Assessment	Reference Values	0 Months (Baseline)	3 Months	6 Months	9 Months	12 Months
Vitamin D (ng/mL)	30–100	24.15 (4.8–64.7)	32.75 (8.8–84.4)	40.8 (14.5–124.0)	49.0 (24.4–89.3)	48.1 (22.0–76.7)
Cortisol (µg/dL)	4.82–19.5	14.1 (4.0–39.0)	12.1 (3.0–33.0)	11.9 (3.0–26.0)	13.4 (5.0–25.0)	14.0 (5.0–31.0)
TSH (µIU/mL)	0.51–4.3	1.6 (0.2–8.1)	1.6 (0.05–5.9)	1.5 (0.2–4.2)	1.5 (0.02–11.0)	1.7 (0.04–4.8)
T3 (pg/mL)	2.6–5	3.2 (2.2–4.4)	3.0 (2.0–4.5)	3.2 (2.4–5.9)	3.1 (1.9–5.3)	3.2 (2.3–4.6)
T4 (ng/dL)	0.93–1.7	1.3 (1.0–8.0)	1.2 (0.9–2.1)	1.2 (0.9–2.0)	1.2 (0.7–2.0)	1.2 (0.8–1.9)
HbA1c (%)	4–6	5.65 (4.7–8.0)	5.6 (4.2–7.9)	5.4 (3.8–7.9)	5.5 (4.7–8.3)	5.5 (4.2–8.5)
Calcium (mmol/L)	2.1–2.6	2.4 (2.0–2.6)	2.4 (2.1–2.6)	2.4 (1.9–2.7)	2.4 (2.2–2.8)	2.4 (2.1–2.7)
Magnesium (mmol/L)	0.66–1.07	0.83 (0.6–1.0)	0.82 (0.6–2.3)	0.82 (0.3–0.9)	0.82 (0.5–0.9)	0.82 (0.51–0.94)
Selenium (µg/L)	50–120	81.45 (44.4–123.2)	78.7 (41.9–139.3)	84.5 (47.2–143.1)	82.4 (40.7–210.5)	84.3 (38.4–138.7)
Iron (µg/dL)	33–193	97.5 (19.0–212.0)	83.0 (26.0–264.0)	80.5 (14.0–185.0)	89.5 (32.0–197.0)	87.0 (7.2–255.0)
LDH (U/L)	0–289	214.0 (147.0–440.0)	221.0 (163.0–779.0)	222.5 (154.0–477.0)	216.0 (160.0–521.0)	217.0 (149.0–336.0)
Lipase (U/L)	13–60	35.0 (14.0–144.0)	30.5 (11.0–74.0)	32.5 (15.0–77.0)	33.0 (14.0–81.0)	34.0 (15.0–101.0)
LDL-Cholesterol (mg/dL)	<116	127.0 (61.0–251.0)	128.0 (68.0–251.0)	134.5 (61.0–246.0)	125.5 (47.0–265.0)	126.0 (45.0–269.0)
HDL-Cholesterol (mg/dL)	>45	64.0 (32.0–103.0)	56.5 (32.0–103.0)	61.0 (30.0–136.0)	64.0 (34.0–145.0)	63.0 (34.0–105.0)
VLDL-Cholesterol (mg/dL)	<30	10.0 (0–77.0)	12.0 (0–110.0)	12.0 (0–62.0)	13.0 (1.0–58.0)	13.0 (0–66.0)
Triglyceride (mg/dL)	<150	103.0 (38.0–525.0)	112.5 (45.0–531.0)	117.5 (44.0–441.0)	112.5 (43.0–387.0)	115.0 (47.0–424.0)
CA 15-3 (U/mL)	<26.2	18.6 (6.2–79.9)	22.3 (6.1–58.2)	19.9 (5.2–46.1)	16.3 (6.4–37.5)	17.3 (6.5–38.2)

**Table 5:** Eating habits. The following foods were consumed by the patients per month, including the vitamin D concentration of these foods. As all values were not normally distributed by Kolmogorov–Smirnov test, all values are given as median with minimum and maximum value.

Food	Median (Min–Max)	Vitamin D Content (μg per 100 g) *
Herring/trout/salmon	3.0 (0–12)	7.80–25.00
Mackerel/tuna	1.0 (0–8)	4.00
Eggs/margarine	12.0 (0–28)	2.50–7.50
Cream/gouda/butter	20.0 (0–28)	1.20–1.30
Whole milk/quark/yogurt	20.0 (0–28)	0.09
Chanterelles/champignons/porcini mushrooms	3.0 (0–28)	1.90–2.10
Beef/veal liver	0 (0–5)	0.33–1.70

* data source: [23].

**Table 6:** GEE analysis. Association of Vitamin D serum levels (ng/mL) with other parameters throughout the year (0, 3, 6, 9, 12 months). Therefore, univariable GEE analysis was performed first. All statistically significant variables have been incorporated in a multiple GEE analysis. Multiple GEE analysis was then repeated to prove accuracy. The following variables provided statistically significant results in univariable GEE analysis and were therefore incorporated in the multiple GEE analysis: Vitamin D substitution, season of assessment, age, weight, BMI, body fat, muscle mass, LDL, VLDL, TSH, T4, calcium, magnesium, selenium, radiotherapy, endocrine therapy, Her2 targeted therapy, CA 15-3, and polyneuropathy. Values that provided statistically significant association with vitamin D serum levels after two multiple GEE analysis are highlighted in bold.

**Lifestyle Variables**			
	**Regression Coefficient**	**95% Confidence Interval**	***p*-Value**
Amount of vitamin D substitution (IU/week)	0.001	(0; 0.001)	**<0.001**
Season of assessment			**<0.001** (all seasons)
Spring	3.19	(0.64, 5.73)	0.01
Summer	6.66	(3.70, 9.62)	**<0.001**
Autumn	4.74	(2.11, 7.37)	**<0.001**
(Winter was used as reference)			
Age	0.15	(0.01, 0.28)	**0.03**
Weight	0.35	(−0.63, 1.33)	0.49
BMI	−0.71	(−1.57, 0.15)	0.11
Body composition:			
Body fat	−0.27	(−1.28, 0.75)	0.61
Muscle mass	−0.52	(−1.98, 0.94)	0.49
Bone mass	−3.90	(−9.24, 1.44)	0.15
Visceral fat	−0.48	(−1.07, 0.12)	0.12
Cortisol	0.06	(−0.24, 0.35)	0.71
LDL	−0.04	(−0.08, −0.01)	0.09
HDL	0.11	(−0.03, 0.24)	0.11
VLDL	−0.15	(−0.22, −0.08)	**<0.001**
HbA1c	−1.43	(−4.97, 2.11)	0.43
TSH	0.16	(−0.88, 1.20)	0.77
T3	1.25	(−2.78, 5.28)	0.54
T4	0.55	(−6.12, 7.21)	0.87
Minerals			
Calcium	8.62	(−1.63, 18.88)	0.10
Magnesium	−9.75	(−16.65, −2.84)	**0.006**
Trace elements			
Iron	−0.02	(−0.06, 0.02)	0.35
Selenium	−0.01	(−0.12, 0.11)	0.91
**Antineoplastic Therapy Related Factors**			
	**Regression Coefficient**	**95% Confidence Interval**	***p*-Value**
Chemotherapy	1.41	(−1.42, 4.24)	0.33
Radiotherapy	−0.33	(−2.78; 2.11)	0.79
Endocrine therapy			**<0.001**
Tamoxifen	3.35	(−1.10; 7.79)	0.14
Aromatase inhibitor	8.58	(5.17; 11.99)	**<0.001**
(no endocrine therapy served as reference)			
Her2 targeted therapy	−2.05	(−5.49; 1.40)	0.24
**Cancer and Side Effect Related Factors**			
	**Regression Coefficient**	**95% Confidence Interval**	***p*-Value**
Tumor marker (CA 15−3)	−0.08	(−0.21, 0.06)	0.25
Polyneuropathy			
CTC grade 0	−4.24	(−11.07, 2.58)	0.22
CTC grade 1	−2.35	(−8.98, 4.27)	0.49
CTC grade 2	0.24	(−5.87, 6.35)	0.94
(CTC grade 3 served as reference)			

**Table 7:** Linear regression. Some values were only available at baseline visit, therefore linear regression was carried out to analyze an association between baseline vitamin D serum levels and possible influencing factors. For this analysis, patients who substituted vitamin D were excluded for the analysis regarding eating habits. Linear regression was first performed in univariate. As only cream/gouda/butter provided a statistically significant result, no multiple linear regression was performed.

Lifestyle Variables *			
**Dietary Habits**	**Regression Coefficient**	**95% Confidence Interval**	***p*-Value**
Herring/trout/salmon	0.26	(−0.74, 1.26)	0.61
Mackerel/tuna	−0.46	(−1.75, 0.83)	0.48
Eggs/margarine	0.11	(−0.25, 0.47)	0.55
Cream/gouda/butter	−0.38	(−0.62, −0.14)	0.003
Whole milk/quark/yoghurt	0.004	(−0.29, 0.29)	0.98
Chanterelles/champignons/porcini mushrooms	0.06	(−0.55, 0.66)	0.85
Beef/veal liver	−1.06	(−3.75, 1.64)	0.44
**Cancer/Antineoplastic Therapy Related Factors**			
	**Regression Coefficient**	**95% Confidence Interval**	***p*-Value**
Genetic mutation	2.63	(−6.5, 11.8)	0.57

* Only patients without vitamin D substitution at baseline were considered for the analysis.

## Data Availability

The data presented in this study are available on request from the corresponding author. The data are not publicly available yet, as further analysis is still ongoing.

## Supplementary Materials

The following supporting information can be downloaded at: https://www.mdpi.com/article/10.3390/nu16060854/s1, Figure S1: Vitamin D serum levels throughout the year (0, 3, 6, 9, 12 months), considering the seasons and substitution of vitamin D.

## Abbreviations

25(OH)D	Serum 25-hydroxyvitamin D
BMI	Body mass index
HER2	Human epidermal growth factor 2
GEE	Generalized estimating equations analysis
CTC	Common toxicity criteria

## References

[1] World Health Organization Obesity Report. https://Www.Who.Int/Data/Gho/Indicator-Metadata-Registry/Imr-Details/3420#:~:Text=The%20relationship%20between%20poor%20healthCaused%20by%20overweight%20or%20obesity.

[2] Gröber U., Holzhauer P., Kisters K., Holick M.F., Adamietz I.A. (2016). Micronutrients in Oncological Intervention. Nutrients.

[3] Hintzpeter B., Mensink G.B.M., Thierfelder W., Müller M.J., Scheidt-Nave C. (2008). Vitamin D Status and Health Correlates among German Adults. Eur. J. Clin. Nutr..

[4] Linseisen J., Bechthold A., Bischoff-Ferrari H., Hinzpeter B., Leschik-Bonnet E., Reichrath J., Stehle P., Volkert D., Wolfram G., Zittermann A. (2011). Vitamin D und Prävention Ausgewählter Chronischer Krankheiten.

[5] Amrein K., Scherkl M., Hoffmann M., Neuwersch-Sommeregger S., Köstenberger M., Tmava Berisha A., Martucci G., Pilz S., Malle O. (2020). Vitamin D Deficiency 2.0: An Update on the Current Status Worldwide. Eur. J. Clin. Nutr..

[6] Park S.-H., Hoang T., Kim J. (2021). Dietary Factors and Breast Cancer Prognosis among Breast Cancer Survivors: A Systematic Review and Meta-Analysis of Cohort Studies. Cancers.

[7] Shao T., Klein P., Grossbard M.L. (2012). Vitamin D and Breast Cancer. Oncologist.

[8] Vanhevel J., Verlinden L., Doms S., Wildiers H., Verstuyf A. (2022). The Role of Vitamin D in Breast Cancer Risk and Progression. Endocr. Relat. Cancer.

[9] de La Puente-Yagüe M., Cuadrado-Cenzual M.A., Ciudad-Cabañas M.J., Hernández-Cabria M., Collado-Yurrita L. (2018). Vitamin D: And Its Role in Breast Cancer. Kaohsiung J. Med. Sci..

[10] Zemlin C., Altmayer L., Stuhlert C., Schleicher J.T., Wörmann C., Lang M., Scherer L.-S., Thul I.C., Spenner L.S., Simon J.A. (2023). Prevalence and Relevance of Vitamin D Deficiency in Newly Diagnosed Breast Cancer Patients: A Pilot Study. Nutrients.

[11] National Cancer Institute Cancer Stat Facts: Female Breast Cancer Subtypes. https://seer.cancer.gov/statfacts/html/breast-subtypes.html.

[12] Xu J., Cao B., Li C., Li G. (2023). The Recent Progress of Endocrine Therapy-Induced Osteoporosis in Estrogen-Positive Breast Cancer Therapy. Front. Oncol..

[13] Deroo B.J. (2006). Estrogen Receptors and Human Disease. J. Clin. Investig..

[14] Nisha Y., Dubashi B., Bobby Z., Sahoo J.P., Kayal S., Ananthakrishnan R., Ganesan P. (2023). Cytotoxic Chemotherapy Is Associated with Decreased Bone Mineral Density in Postmenopausal Women with Early and Locally Advanced Breast Cancer. Arch. Osteoporos..

[15] Jennaro T.S., Fang F., Kidwell K.M., Smith E.M.L., Vangipuram K., Burness M.L., Griggs J.J., Van Poznak C., Hayes D.F., Henry N.L. (2020). Vitamin D Deficiency Increases Severity of Paclitaxel-Induced Peripheral Neuropathy. Breast Cancer Res. Treat..

[16] Holick M.F. (2007). Vitamin D Deficiency. N. Engl. J. Med..

[17] Kok D.E., van den Berg M.M.G.A., Posthuma L., van ’t Erve I., van Duijnhoven F.J.B., de Roos W.K., Grosfeld S., Los M., Sommeijer D.W., van Laarhoven H.W.M. (2019). Changes in Circulating Levels of 25-Hydroxyvitamin D3 in Breast Cancer Patients Receiving Chemotherapy. Nutr. Cancer.

[18] Kim H.J., Koh B.S., Yu J.H., Lee J.W., Son B.H., Kim S.B., Ahn S.H. (2014). Changes in Serum Hydroxyvitamin D Levels of Breast Cancer Patients during Tamoxifen Treatment or Chemotherapy in Premenopausal Breast Cancer Patients. Eur. J. Cancer.

[19] Bleizgys A. (2021). Vitamin D Dosing: Basic Principles and a Brief Algorithm (2021 Update). Nutrients.

[20] Zemlin C., Stuhlert C., Schleicher J.T., Wörmann C., Altmayer L., Lang M., Scherer L.S., Thul I.C., Müller C., Kaiser E. (2021). Longitudinal Assessment of Physical Activity, Fitness, Body Composition, Immunological Biomarkers, and Psychological Parameters during the First Year after Diagnosis in Women with Non-Metastatic Breast Cancer: The BEGYN Study Protocol. Front. Oncol..

[21] Hardin J.W., Hilbe J.M. (2002). Generalized Estimating Equations.

[22] National Cancer Institute, Division of Cancer Treatment & Diagnosis, Common Terminology Criteria for Adverse Events (CTCAE). https://ctep.cancer.gov/.

[23] Souci S.W., Fachmann W., Kraut H., Andersen G. (2008). Souci/Fachmann/Kraut Food Composition and Nutrition.

[24] Cashman K.D., Dowling K.G., Škrabáková Z., Gonzalez-Gross M., Valtueña J., De Henauw S., Moreno L., Damsgaard C.T., Michaelsen K.F., Mølgaard C. (2016). Vitamin D Deficiency in Europe: Pandemic?. Am. J. Clin. Nutr..

[25] Schweizer A.-M., Leiderer A., Mitterwallner V., Walentowitz A., Mathes G.H., Steinbauer M.J. (2021). Outdoor Cycling Activity Affected by COVID-19 Related Epidemic-Control-Decisions. PLoS ONE.

[26] Park A.H., Zhong S., Yang H., Jeong J., Lee C. (2022). Impact of COVID-19 on Physical Activity: A Rapid Review. J. Glob. Health.

[27] (2023). AGO Mamma Guidelines Breast Version 2023.1E Adjuvant Endocrine-Based Therapy in Pre- and Postmenopausal Patients. www.ago-online.de.

[28] Cardoso F., Kyriakides S., Ohno S., Penault-Llorca F., Poortmans P., Rubio I.T., Zackrisson S., Senkus E. (2019). Early Breast Cancer: ESMO Clinical Practice Guidelines for Diagnosis, Treatment and Follow-Up. Ann. Oncol..

[29] Holick M.F., Binkley N.C., Bischoff-Ferrari H.A., Gordon C.M., Hanley D.A., Heaney R.P., Murad M.H., Weaver C.M. (2011). Evaluation, Treatment, and Prevention of Vitamin D Deficiency: An Endocrine Society Clinical Practice Guideline. J. Clin. Endocrinol. Metab..

[30] Pludowski P., Takacs I., Boyanov M., Belaya Z., Diaconu C.C., Mokhort T., Zherdova N., Rasa I., Payer J., Pilz S. (2022). Clinical Practice in the Prevention, Diagnosis and Treatment of Vitamin D Deficiency: A Central and Eastern European Expert Consensus Statement. Nutrients.

[31] Maeda S.S., Kunii I.S., Hayashi L., Lazaretti-Castro M. (2007). The Effect of Sun Exposure on 25-Hydroxyvitamin D Concentrations in Young Healthy Subjects Living in the City of São Paulo, Brazil. Braz. J. Med. Biol. Res..

[32] Wortsman J., Matsuoka L.Y., Chen T.C., Lu Z., Holick M.F. (2000). Decreased Bioavailability of Vitamin D in Obesity. Am. J. Clin. Nutr..

[33] Kweder H., Eidi H. (2018). Vitamin D Deficiency in Elderly: Risk Factors and Drugs Impact on Vitamin D Status. Avicenna J. Med..

[34] MacLaughlin J., Holick M.F. (1985). Aging Decreases the Capacity of Human Skin to Produce Vitamin D3. J. Clin. Investig..

[35] Levin G.P., Robinson-Cohen C., de Boer I.H., Houston D.K., Lohman K., Liu Y., Kritchevsky S.B., Cauley J.A., Tanaka T., Ferrucci L. (2012). Genetic Variants and Associations of 25-Hydroxyvitamin D Concentrations With Major Clinical Outcomes. JAMA.

[36] Matsui M.S. (2020). Vitamin D Update. Curr. Dermatol. Rep..

[37] Poskitt E.M.E., Cole T.J., Lawson D.E.M. (1979). Diet, Sunlight, and 25-Hydroxy Vitamin D in Healthy Children and Adults. Br. Med. J..

[38] Heidari B., Mirghassemi M.B.H. (2012). Seasonal Variations in Serum Vitamin D According to Age and Sex. Casp. J. Intern. Med..

[39] Franca Gois P., Wolley M., Ranganathan D., Seguro A. (2018). Vitamin D Deficiency in Chronic Kidney Disease: Recent Evidence and Controversies. Int. J. Environ. Res. Public Health.

[40] Nowak J., Jabczyk M., Jagielski P., Hudzik B., Brukało K., Borszcz J., Zubelewicz-Szkodzińska B. (2023). Could Vitamin D Concentration Be a Marker of a Long Hospital Stay in Older Adults Patients?. Front. Nutr..

[41] Harris S.S., Dawson-Hughes B. (2002). Plasma Vitamin D and 25OHD Responses of Young and Old Men to Supplementation with Vitamin D. J. Am. Coll. Nutr..

[42] Clemens T.L., Zhouf X.-Y., Myles M., Endres D., Linsay R. (1986). Serum Vitamin D2 and Vitamin D3 Metabolite Concentrations and Absorption of Vitamin D2 in Elderly Subjects. J. Clin. Endocrinol. Metab..

[43] Fitzgerald J.S., Swanson B.J., Larson-Meyer D.E. (2023). Vitamin D Knowledge, Awareness, and Attitudes of Adolescents and Adults: A Systematic Review. J. Nutr. Educ. Behav..

[44] Borecka O., Farrar M.D., Osman J.E., Rhodes L.E., Webb A.R. (2021). Older Adults Who Spend More Time Outdoors in Summer and Have Higher Dietary Vitamin D than Younger Adults Can Present at Least as High Vitamin D Status: A Pilot Study. Int. J. Environ. Res. Public Health.

[45] Gallagher J.C., Yalamanchili V., Smith L.M. (2013). The Effect of Vitamin D Supplementation on Serum 25OHD in Thin and Obese Women. J. Steroid Biochem. Mol. Biol..

[46] Kim M.R., Jeong S.J. (2019). Relationship between Vitamin D Level and Lipid Profile in Non-Obese Children. Metabolites.

[47] Uwitonze A.M., Razzaque M.S. (2018). Role of Magnesium in Vitamin D Activation and Function. J. Am. Osteopath. Assoc..

[48] Cheung M.M., Dall R.D., Shewokis P.A., Altasan A., Volpe S.L., Amori R., Singh H., Sukumar D. (2022). The Effect of Combined Magnesium and Vitamin D Supplementation on Vitamin D Status, Systemic Inflammation, and Blood Pressure: A Randomized Double-Blinded Controlled Trial. Nutrition.

[49] Dai Q., Zhu X., Manson J.E., Song Y., Li X., Franke A.A., Costello R.B., Rosanoff A., Nian H., Fan L. (2018). Magnesium Status and Supplementation Influence Vitamin D Status and Metabolism: Results from a Randomized Trial. Am. J. Clin. Nutr..

[50] Goldvaser H., Barnes T.A., Šeruga B., Cescon D.W., Ocaña A., Ribnikar D., Amir E. (2018). Toxicity of Extended Adjuvant Therapy With Aromatase Inhibitors in Early Breast Cancer: A Systematic Review and Meta-Analysis. J. Natl. Cancer Inst..

[51] Krishnan A.V., Swami S., Peng L., Wang J., Moreno J., Feldman D. (2010). Tissue-Selective Regulation of Aromatase Expression by Calcitriol: Implications for Breast Cancer Therapy. Endocrinology.

[52] Al Khalifah R., Alsheikh R., Alnasser Y., Alsheikh R., Alhelali N., Naji A., Al Backer N. (2020). The Impact of Vitamin D Food Fortification and Health Outcomes in Children: A Systematic Review and Meta-Regression. Syst. Rev..

[53] Nikooyeh B., Neyestani T.R. (2022). The Effects of Vitamin D-Fortified Foods on Circulating 25(OH)D Concentrations in Adults: A Systematic Review and Meta-Analysis. Br. J. Nutr..

[54] Niedermaier T., Gredner T., Kuznia S., Schöttker B., Mons U., Lakerveld J., Ahrens W., Brenner H. (2022). Vitamin D Food Fortification in European Countries: The Underused Potential to Prevent Cancer Deaths. Eur. J. Epidemiol..

[55] Cui A., Xiao P., Ma Y., Fan Z., Zhou F., Zheng J., Zhang L. (2022). Prevalence, Trend, and Predictor Analyses of Vitamin D Deficiency in the US Population, 2001–2018. Front. Nutr..

[56] Marcinowska-Suchowierska E., Kupisz-Urbańska M., Łukaszkiewicz J., Płudowski P., Jones G. (2018). Vitamin D Toxicity—A Clinical Perspective. Front. Endocrinol..

[57] Pellegrino G., Ascenti V., Desiderio E., Carrafiello G. (2023). Vitamin D Intoxication: Myth or Reality. Minerva Med..

[58] Šimoliūnas E., Rinkūnaitė I., Bukelskienė Ž., Bukelskienė V. (2019). Bioavailability of Different Vitamin D Oral Supplements in Laboratory Animal Model. Medicina.

