# Supplementary material for: Course of Vitamin D Levels in Newly Diagnosed Non-Metastatic Breast Cancer Patients over One Year with Quarterly Controls and Substitution
Source: Nutrients. 2024 Mar 15;16(6):854. doi: 10.3390/nu16060854 (PMC10975236; doi:10.3390/nu16060854)
Supplement: Supplementary file 1 [file nutrients-16-00854-s001.zip › nutrients-2896732-supplementary.pdf]

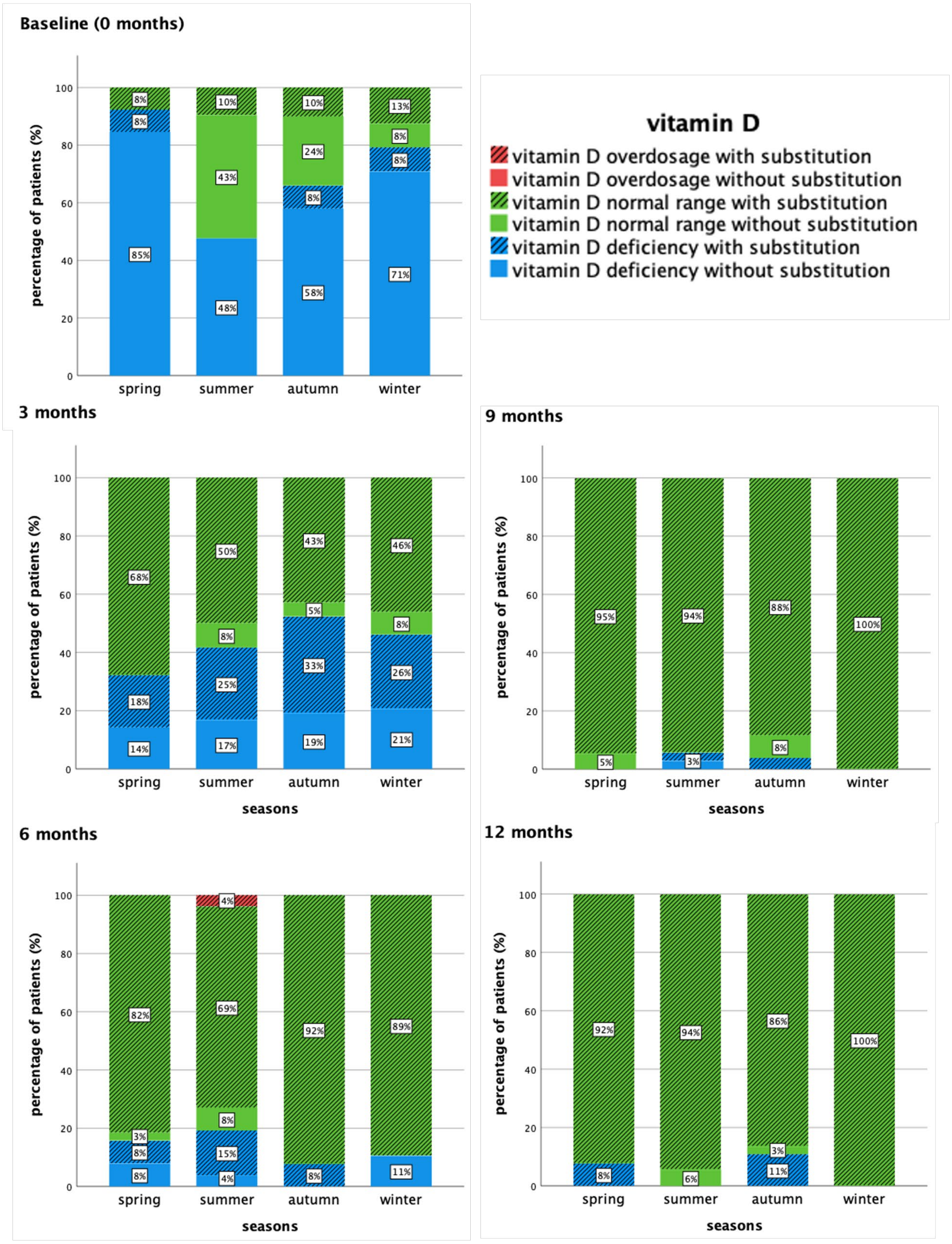

Supplementary Figure S1. Vitamin D serum levels throughout the year (0, 3, 6, 9, 12 months), considering the seasons and substitution of vitamin D.
